# Prevalence and risk factors of thrombosis in patients with congenital hyperinsulinism: a retrospective analysis

**DOI:** 10.3389/fendo.2025.1611224

**Published:** 2025-07-09

**Authors:** Mohammed Hady Albitar, Nida Mariyam, Ziad Alhosainy, Raghad Alhuthil, Marah Nayfeh, Maeen Aldamouni, Seba Albitar, Afaf Alsagheir

**Affiliations:** ^1^ College of Medicine, Alfaisal University, Riyadh, Saudi Arabia; ^2^ Department of Pediatrics, King Faisal Specialist Hospital and Research Centre, Riyadh, Saudi Arabia

**Keywords:** congenital hyperinsulinism (CHI), thrombosis, central venous catheter (CVC), *ABCC8* gene, *USH1C*, Saudi Arabia

## Abstract

**Background:**

Congenital hyperinsulinism (CHI) is a rare but significant cause of persistent neonatal hypoglycemia. While Central Venous Catheters (CVCs) are a known major risk factor for thrombosis in neonates, the evidence linking CHI, catheter use, and thrombotic risk remains limited. This study investigates the prevalence of thrombosis in CHI patients and explores potential contributing factors, such as CVC insertion and infection.

**Methods:**

A retrospective cohort study was conducted on 67 patients under 14 years of age who were diagnosed with CHI and treated at King Faisal Specialist Hospital and Research Centre in Riyadh, Saudi Arabia, between 2014 and 2024. Clinical, genetic, and imaging data were analyzed, and associations between thrombosis and risk factors were assessed using univariable analysis.

**Results:**

Of the 67 patients with CHI, 53.7% were female, with a median age at CHI diagnosis of 3 weeks. Genetic analysis revealed *ABCC8* mutations as the most frequently identified genetic variant (58.2%). CVCs were used in 61 cases (91.0%), with thrombosis developing in 18.0% of those with CVCs, mostly affecting the vena cava and portal vein. All thrombosis cases were treated with enoxaparin; 63.6% of patients experienced mild, transient complications, including elevated liver enzymes, prolonged partial thromboplastin time (PTT), and thrombocytopenia. A statistically significant association was found between infection and thrombosis (p = 0.001), but no significant correlation was found between specific genetic mutations and thrombosis risk (p > 0.05).

**Conclusion:**

These findings underscore the importance of recognizing thrombosis as a potential complication in patients with CHI undergoing CVC placement. Although most cases were successfully managed, early screening and preventive strategies should be considered to minimize thrombotic complications. Future research should focus on optimizing thromboprophylaxis and evaluating additional contributing factors to refine management strategies and improve patient outcomes.

## 1 Introduction

Congenital hyperinsulinism (CHI) is a rare yet notable condition that causes persistent and severe hypoglycemia in neonates and infants due to excessive insulin secretion. The estimated prevalence is approximately 1 in 40,000 live births, with a higher incidence in consanguineous populations (~1 in 2,500) (1). This condition is caused by genetic defects in pancreatic beta cells, primarily involving mutations in ATP-sensitive potassium channel genes, leading to unregulated insulin secretion and an increased glucose demand (2). Despite advancements in molecular diagnostics and therapeutic approaches, CHI remains a high-impact condition, with long-term neurodisability rates ranging from 26% to 48% across various cohorts (3–5). Neonates with CHI often present with high birth weight and increased glucose utilization, necessitating initial glucose support through intravenous infusions of high concentrations of dextrose via a central venous catheter (CVC) (6). While critical for maintaining optimal glucose levels, CVC insertion increases the risk of both infection and thrombosis (7).

CVCs are a well-established risk factor for thrombosis in hospitalized children, with a recent systematic review and meta-analysis reporting pooled incidence rates between 1.6% and 38% depending on the patient population and catheter type (8). While CVCs remain a major risk factor for thrombosis in neonates, limited research has focused on the specific risk of thrombosis in patients with CHI, although few studies have examined CVC-associated thrombosis in pediatric populations, including patients with CHI (8–10). These studies identified a significant incidence of thrombosis but left questions regarding genetic predisposition and potential preventive strategies. This gap is notable given the complex relationship between the hyperinsulinemic state, the hemostatic system, and the reliance on prolonged CVC use in these patients (8). This study examines thrombosis rates in patients with CHI and evaluates its association with CVC insertion and other contributing factors, offering a more comprehensive understanding of thrombotic risk in this population.

## 2 Methods

A retrospective cohort study of patients with CHI was conducted at King Faisal Specialist Hospital and Research Centre, Riyadh, Saudi Arabia. The study included pediatric patients under 14 years of age with a confirmed CHI diagnosis and attended follow-up visits between 2014 and 2024. All enrolled patients underwent both echocardiography and abdominal ultrasonography.

Data were collected through chart review for all eligible patients, including demographic information, imaging findings (echocardiogram and abdominal ultrasound/Doppler), genetic test results, clinical interventions (e.g., CVC insertion), laboratory findings, and patient outcomes. Relevant information aligned with the study objectives was extracted from electronic medical records. The data were presented systematically using tables and graphs. In all cases, CVCs were primarily used for the infusion of high concentration (20%) dextrose solutions to manage persistent hypoglycemia associated with CHI.

Statistical analyses were performed using STATA version 18. Univariable analysis was done using the Fisher exact test. A p-value of less than 0.05 was considered statistically significant. This research was performed according to the guidelines of the Declaration of Helsinki and approved by the Office of Research Affairs at King Faisal Specialist Hospital and Research Centre (Reference number: 2191250). This was a retrospective observational study; therefore, informed consent was not required.

## 3 Results

**Figure 1:**
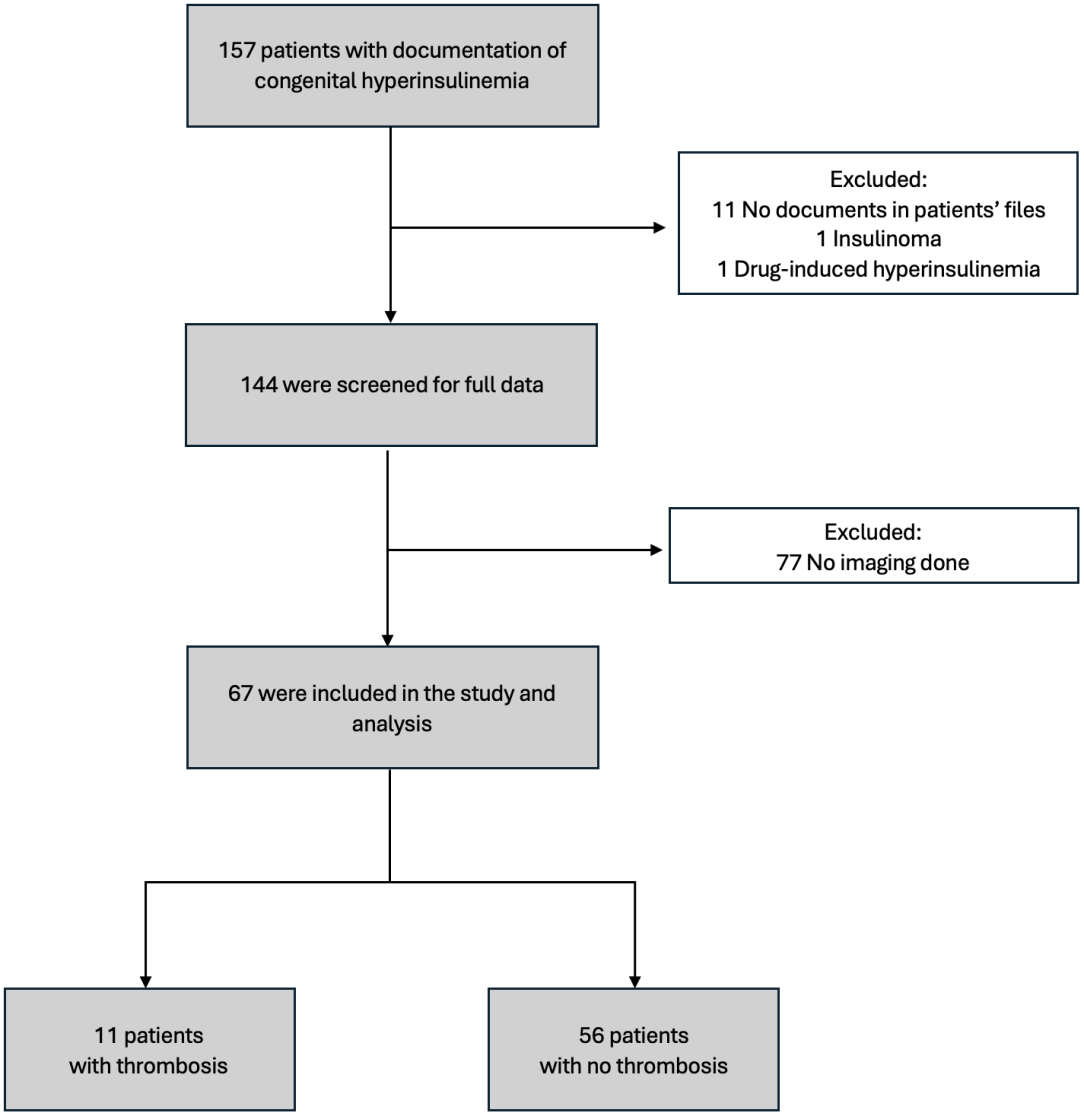
Flowchart of the study cohort. A total of 157 patients with congenital hyperinsulinism were initially identified. After exclusions, 67 patients were included in the study. Among them, 11 patients had documented thrombosis, while 56 patients did not have thrombosis.

**Figure 1** summarizes the distribution of the patients included in the study. Of the 157 patients initially identified with CHI, 67 met the eligibility criteria after exclusions were applied. Among these patients, 53.7% were female and 46.3% were male, with a median age of 3 weeks at the time of diagnosis. Laboratory evaluations performed during hypoglycemic episodes (glucose <2.7 mmol/L) showed elevated insulin and C-peptide levels, with median levels of 100.7 pmol/L [46.6–262.3] and 1.08 nmol/L [0.56–1.95], respectively (**Table 1**). Diagnostic tests, including liver function tests, renal function tests, serum bicarbonate, growth hormone, cortisol, lactate, plasma acylcarnitine, and urine organic acids were unremarkable. Notably, free fatty acids (FFA) and beta-hydroxybutyrate (BOHB) levels were low in all patients. 54 patients (80.6%) underwent near-total pancreatectomy. Blood cultures were positive in 18 patients (28.4%), and bacterial infections, including *Klebsiella pneumoniae* and *Staphylococcus aureus* were the most common pathogens (**Table 1**). Univariable analysis revealed a statistically significant association between infection and thrombosis, with 72.7% of patients who developed thrombosis having an infection, compared to 19.6% of those without thrombosis (p = 0.001) (**Table 2**).

**Table 1:** Characteristics of patients with congenital hyperinsulinism (n = 67).

Characteristics	N (%), Median [IQR]
Gender	
-Female	36 (53.7)
-Male	31 (46.3)
*Age at CHI diagnosis (weeks)*	3 [birth−8 weeks]
Laboratory investigations	
-Insulin level (pmol/L)	100.7 [46.6−262.3]
-C-peptide (nmol/L)	1.08 [0.56−1.95]
*Undergone near total pancreatectomy*	*54 (80.6)*
Positive blood culture*	19 (28.4)
*-*Bacterial Infection	18 (94.7)
-Fungal Infection	1 (5.3)
Genes identified	
-ABCC8	39 (58.2)
-KCNJ11	10 (14.9)
- ABCC8-USH1C deletion	9 (13.4)
-GCK	3 (4.5)
-HNF4A	3 (4.5)
-GLUD1	2 (3.0)
-HID1	1 (1.5)

CHI, Congenital Hyperinsulinism; IQR, Interquartile Range;

*Blood cultures identified the following organisms, *Klebsiella pneumoniae, Staphylococcus aureus (including MRSA), Enterococcus faecium, Enterobacter cloacae complex, Staphylococcusepidermidis, Candida albicans, Escherichia coli (E. coli)*.

**Table 2:** Factors associated with thrombosis.

Parameters	Thrombosis (N = 11)	No thrombosis (N = 56)	*P*
Males	7 (63.6)	24 (42.9)	0.322
ABCC8	5 (45.5)	34 (60.7)	0.505
KCNJ11	9 (16.1)	1 (9.1)	1.000
ABCC8-USH1C deletion	3 (27.3)	6 (10.7)	0.159
Central venous catheter (CVC) insertion	11 (100)	50 (89.3)	0.579
Infection	8 (72.7)	11 (19.6)	0.001*

*Indicates statistical significance.

Genetic analysis revealed *ABCC8* mutations as the most common variant identified (58.2%), followed by *KCNJ11* mutations (14.9%) and *ABCC8-USH1C* deletions (13.4%) (**Table 1**). However, no statistically significant correlation was found between specific genetic mutations and thrombosis risk (p > 0.05) (**Table 2**).

CVC insertion was documented in 61 cases (91.0%). Among these, thrombosis occurred in 11 cases (18%). The median age at the onset of thrombosis was 4 weeks. The most common sites for thrombosis were the vena cava (6 patients) and the portal vein (4 patients) (**Table 3A**). Imaging revealed thrombotic abnormalities in 8 cases (11.9%) on echocardiography and in 9 cases (13.4%) on abdominal ultrasound/Doppler. Notably, 6 patients (9.0%) showed abnormalities on both imaging modalities (**Table 3A**). The echocardiograms and/or abdominal ultrasounds were performed when there was clinical suspicion of sepsis, catheter malfunction, or limb swelling.

**Table 3a:** CVC insertion and thrombosis characteristics.

Characteristics	N (%), Median [IQR]
*Central venous catheter (CVC) insertion*	61 (91.0)
*Thrombosis*	11 (18.0)
-Venous thrombosis	8 (72.7)
-Arterial thrombosis	1 (9.1)
-Both arterial and venous thrombosis	2 (18.2)
Thrombosis site*	
*Arterial:*	
-Right pulmonary artery	2 (18.2)
-Left atrium	1 (9.1)
*Venous:*	
-Inferior vena cava	6 (54.5)
-Portal veins	4 (36.4)
-Left internal jugular	3 (27.3)
-Left subclavian	2 (18.2)
-Right internal jugular	2 (18.2)
-Superior vena cava	1 (9.1)
-Right femoral	1 (9.1)
-Left innominate	1 (9.1)
-Left brachial	1 (9.1)
-Left axillary	1 (9.1)
-Bilateral common iliac	1 (9.1)
-Hepatic veins	1 (9.1)
*Median Age at thrombosis (weeks)*	4 [1−8]
*Enoxaparin (all thrombosis cases)*	11/11 (100.0)
Enoxaparin-Related Complications (7 out of 11 cases)**	*7 (63.6)*
-Elevated Liver Enzymes	3 (42.9)
-Prolonged Partial Thromboplastin Time (PTT)	3 (42.9)
-Bleeding	1 (14.3)
-Thrombocytopenia	2 (28.6)
*Time to Thrombosis Resolution (days)*	72 [60−90]

*Multiple thrombosis sites were observed in some patients.

**One patient experienced three complications related to Enoxaparin: elevated liver enzymes, prolonged PTT, and thrombocytopenia.

**Table 3b:** Clinical characteristics of patients with multi-site thrombosis (n=8).

No	Gender	Sites of thrombosis	Blood culture	Genetic mutation	Coagulation Factor Deficiency
1	Male	1- Right femoral vein 2- Left internal jugular vein 3- Left subclavian vein 4- Left brachial vein 5- Left axillary vein	Positive	ABCC8-USH1C deletion	Normal
2	Female	1-Right internal jugular vein 2-Left internal jugular vein 3- Left innominate vein	Negative	ABCC8-USH1C deletion	Normal
3	Male	1-Inferior vena cava 2- Left portal vein	Positive	KCNJ11	Normal
4	Male	1- Inferior vena cava 2- Bilateral common iliac veins.	Positive	HNF4A	Normal
5	Male	1-Inferior vena cava 2-Right pulmonary artery 3-Hepatic vein	Negative	ABCC8	Protein S
6	Female	1- Inferior vena cava 2- Right pulmonary artery	Positive	ABCC8	Protein C, Protein S, Anti-thrombin III
7	Female	1-Left portal vein 2- Superior vena cava 3- Left subclavian vein	Positive	ABCC8-USH1C deletion	Normal
8	Female	1- Right internal jugular vein 2- Left internal jugular vein	Positive	HID1	Normal

Three patients with thrombosis had low levels of protein C, one had low protein S, and two had low antithrombin III. However, these deficiencies were generally mild, and in most cases, the levels returned to normal over time. All patients diagnosed with thrombosis (n = 11) received enoxaparin treatment. Of these, 7 patients (63.6%) experienced enoxaparin-related complications, including elevated liver enzymes in 3 patients (42.9%), prolonged partial thromboplastin time in 3 (42.9%), thrombocytopenia in 2 (28.6%), and bleeding in 1 (14.3%) (**Table 3A**). The median duration of thrombosis resolution was approximately 72 days.

**Table 3B** provides a detailed breakdown of thrombosis sites of cases with multi-site involvement. Notably, the vena cava was the most commonly affected site followed by the internal jugular veins. Among these patients, 6 out of 8 (75%) had positive blood cultures, suggesting a potential link between infection and multi-site thrombosis. Additionally, 2 out of 8 (25%) patients with multi-site involvement exhibited abnormal protein C, protein S, or anti-thrombin III levels. However, no consistent correlation was found between these coagulation abnormalities and the thrombosis sites.

## 4 Discussion

This study aimed to assess the prevalence and risk factors of CVC related thrombosis (CVC-RT) in patients with CHI, a rare but significant cause of persistent hypoglycemia in neonates and children, resulting from dysregulated insulin secretion by pancreatic beta cells (1, 2). In clinical practice, maintaining blood glucose levels above 70 mg/dL is recommended to minimize the risk of hypoglycemia-induced brain injury (11, 12). CVCs are commonly utilized to administer concentrated glucose solutions in patients with CHI to manage persistent hypoglycemia (13). However, CVC placement is a well-established risk factor for thrombosis in pediatric patients (9). In our study, CVCs were used predominantly for delivering 20% dextrose infusions to manage hypoglycemia in CHI patients.

CVC-RT can arise due to endothelial injury at the catheter site, venous stasis, the prothrombotic state triggered by inflammation, and other catheter-related complications (7, 14). In a case series, Yau et al. (10) reported an 18% incidence of CVC-related thrombosis (CVC-RT) in patients with CHI (10). Our findings similarly showed an 18% incidence of thrombosis, aligning with their results. Although univariate analysis did not show a statistically significant association between CVC insertion and thrombosis (p = 0.579), thrombosis occurred more frequently in patients with CVCs, suggesting a potential association that requires additional studies. The CVC-RT associated thrombosis among pediatric ICU patients is considered lower, at around 5.4% as reported by Azzam et al., further reinforcing the comparatively heightened thrombotic risk in patients with CHI (9). Their study also identified high baseline D-dimer levels and low platelet counts as independent risk factors for CVC-RT (9). In addition, peripherally inserted central catheter use showed a significantly higher thrombosis risk than centrally placed line use (51.9% vs. 29.6%, p = 0.011) (9).

A recent systematic review and meta-analysis by Fu et al. analyzed more than 50 studies and identified key risk factors for CVC-RT, including catheter indwelling time, insertion site (femoral lines carrying the highest risk), multi-lumen catheters, central line-associated bloodstream infections (CLABSI), and high D-dimer levels (8). The strong association between CLABSI and thrombosis found in Fu et al.’s meta-analysis further supports our observed link between infection and thrombosis (p = 0.001), offering insights that may have important implications for the management of CHI in neonates. The most commonly affected sites in our cohort were the vena cava and portal vein, and this reflects the pattern of central line-associated thrombi in neonates (15). CVC-RT complications include venous blockages, reduced future options for vascular access, and the risk of embolic events, all of which may significantly affect the clinical outcomes (16).

Hyperinsulinemia promotes a prothrombotic state through various mechanisms (17). Elevated insulin levels enhance platelet aggregation and increase the secretion of plasminogen activator inhibitor-1, which inhibits fibrinolysis and promotes clot formation (18). This imbalance impairs clot breakdown and elevates thrombosis risk. Hyperinsulinemia also induces endothelial dysfunction by increasing oxidative stress and inflammation in vascular tissues, both of which contribute to thrombosis (17). While these effects are well documented in metabolic disorders such as type 2 diabetes, they are also relevant in CHI, where persistent hyperinsulinemia is a hallmark feature. When combined with the mechanical and inflammatory risks associated with prolonged CVC use, the likelihood of thrombosis in patients with CHI increases, highlighting the need for targeted thromboprophylaxis and close monitoring of these patients, particularly those requiring CVC placement.

In our cohort, homozygous mutations in *ABCC8* and *KCNJ11* were the most frequently observed genetic alterations. However, univariable analysis showed no statistically significant association between these mutations and thrombosis occurrence (p >0.05). Yau et al. (10) reported a significant correlation between homozygous and compound heterozygous *ABCC8/KCNJ11* mutations and an increased risk of thrombosis (R² = 0.40, P = 0.001), suggesting that severe CHI cases carrying these genetic variants may be predisposed to thrombotic events (11). Although HID1 is not classically recognized among the core genes associated with congenital hyperinsulinism (CHI), emerging evidence supports its potential involvement in syndromic forms of the disease (19). Schanzer et al. documented six patients from five unrelated families with biallelic HID1 variants who exhibited combined pituitary hormone deficiency and epileptic encephalopathy (20). Three of these patients also had biochemically confirmed transient hyperinsulinemia, likely influenced by perinatal factors and coexisting hormonal deficiencies, such as growth hormone and cortisol deficiency (20). The proposed pathophysiology involves impaired vesicular trafficking in neuroendocrine cells, which may disrupt insulin release under specific conditions (19, 20). In our current study, we identified a patient with biallelic HID1 variants who presented with biochemically confirmed hyperinsulinism and recurrent hypoglycemia, reinforcing this gene’s potential relevance in syndromic hyperinsulinism, although additional evidence is required to confirm its causative role.

Our genetic analysis did not reveal any abnormalities in coagulation-related genes that could account for the observed cases of thrombosis. Among the 11 patients with thrombosis in our study, three exhibited low levels of protein C, protein S, and/or antithrombin III. However, these deficiencies were not severe, and levels normalized over time in most cases. Additionally, infants younger than six months of age may naturally have lower levels of antithrombin III, protein C, and protein S, as these factors typically take time to reach normal physiological ranges. This suggests that these variations were likely physiological rather than pathological, reinforcing the hypothesis that thrombosis in patients with CHI is primarily driven by hyperinsulinemia rather than an underlying coagulation disorder (21).

Routine ultrasound screening in high-risk CHI patients may aid in the earlier detection and management for thrombosis (2, 9, 10). However, ultrasound screening is not typically performed unless there are clinical indications, such as suspected or documented sepsis (18).

Enoxaparin prophylaxis has been explored as a potential strategy for reducing thrombosis risk in patients with CHI requiring extended CVC use. According to the study by Yau et al. (10), none of the patients with CHI (n = 7) who received enoxaparin prophylaxis developed thrombosis or experienced bleeding complications, suggesting a possible safe protective effect (10). All of the 11 thrombotic cases in our study were managed effectively with enoxaparin. Seven patients receiving enoxaparin experienced at least one complication, including elevated liver enzymes in 3 patients, prolonged partial thromboplastin time in 3 patients, and thrombocytopenia in 2 patients (**Table 3**). Despite this, all cases were successfully managed, and complications were self-limiting. Given the lack of standardized guidelines for anticoagulation in patients with CHI utilizing CVCs, further research is warranted to determine the optimal thromboprophylaxis approach for this high-risk population.

Our study has some limitations. Some key thrombosis risk factors identified in the literature, such as catheter insertion site, number of lumens, and catheter dwell time were not consistently documented and thus could not be reliably analyzed (8). Additionally, although imaging in each case was prompted by clinical suspicion of sepsis, catheter dysfunction or limb swelling, we could not determine the frequency of each specific indication due to the retrospective nature of the study. Despite the promising use of enoxaparin as prophylaxis in CHI requiring CVC, no thromboprophylaxis was given or trialed in our cohort. Lastly, as this was a single-center study from a tertiary referral hospital, the findings may not be generalizable to all CHI populations.

## 5 Conclusion

Thrombosis is an underrecognized significant complication in patients with CHI, particularly among those requiring prolonged CVC use and experiencing concurrent infections. Given the associated morbidity, early screening and risk-mitigation strategies should be prioritized. Further studies are warranted to establish optimal screening and thromboprophylaxis protocols to reduce thrombosis incidence in this vulnerable population.

## Data Availability

The raw data supporting the conclusions of this article will be made available by the authors, without undue reservation.
